# Dietary management practices for type 1 diabetes mellitus by dietitians in KwaZulu-Natal

**DOI:** 10.4102/hsag.v26i0.1506

**Published:** 2021-03-29

**Authors:** Megan E. Dimitriades, Kirthee Pillay

**Affiliations:** 1 Department of Dietetics and Human Nutrition, College of Agriculture, Engineering and Science, University of KwaZulu-Natal, Pietermaritzburg, South Africa

**Keywords:** dietary management practices, dietitians, type 1 diabetes mellitus, dietary guidelines, KwaZulu-Natal

## Abstract

**Background:**

In South Africa, 5% – 15% of diabetics have type 1 diabetes mellitus (T1DM). Dietitians are an important part of the diabetes management team; however, there is a lack of published data on the dietary management practices for T1DM by dietitians.

**Aim:**

The aim of this study was to determine the dietary management practices for T1DM by dietitians in KwaZulu-Natal (KZN).

**Setting:**

This study was conducted in KZN.

**Methods:**

A cross-sectional, descriptive study was conducted using a self-administered electronic questionnaire.

**Results:**

Of the 69 dietitians who participated, 58% (*n* = 40) used the American Diabetes Association (ADA) guidelines to manage T1DM; just under 35% (*n* = 24) spent over an hour with new cases; and 87% (*n* = 60) used face-to-face consultations for follow-up. Dietitians used the glycaemic index, portion control using the healthy eating plate, carbohydrate counting using nutritional labels and household measures and carbohydrate awareness to manage T1DM (*p* < 0.05). Dietitians also used the healthy eating plate (71%; *n* = 49) (*p* < 0.05) and household measures (73.9%; *n* = 51) (*p* < 0.05) to manage T1DM. Time constraints, the literacy level of the patient, available resources and language barriers all played a role in determining the dietary management practices used (*p* < 0.05).

**Conclusion:**

Most dietitians in KZN used the ADA dietary guidelines to manage T1DM, which highlights the need for South African dietary guidelines for the management of T1DM. Dietitians used a variety of different dietary methods to manage T1DM in practice. This suggests that dietitians are flexible in how they manage T1DM with no one particular method being used. A variety of factors also influenced which dietary management practices were chosen.

## Introduction

According to the International Diabetes Federation (IDF), 463 million people worldwide between the ages of 20 and 79 years were living with diabetes mellitus (DM) in 2019. This is estimated to increase to 700 million by 2045 (IDF Diabetes Atlas 2019:34–44). In 2019, 4 million global deaths were attributed to diabetes. Diabetes mellitus is one of the top 10 contributors to deaths worldwide (IDF Diabetes Atlas 2019:54). South Africa has 4.6 million people living with DM (IDF Diabetes Atlas 2019:65:47).

Although type 1 diabetes mellitus (T1DM) has traditionally been associated with children and type 2 diabetes mellitus (T2DM) with adults, this is no longer the case as both types of diabetes can present in either group [American Diabetes Association (ADA) 2021a]. The onset of T1DM is more variable in adults than in children, as an adult may not present with the typical symptoms that children often present with (ADA 2021a). With T1DM, there is autoimmune beta cell destruction leading to absolute insulin deficiency (ADA 2021a). A type 1 diabetic requires daily injections of insulin to maintain an optimal glucose level, and without insulin they would not survive (IDF Diabetes Atlas 2019:13).

The aims of diabetes management are to avoid acute decompensation, prevent or delay complications and mortality, and maintain quality of life (ADA 2021b). The aim of insulin treatment in individuals with T1DM is to obtain as near as normal blood glucose levels to prevent complications associated with diabetes (Hommel et al. 2017). To maintain normoglycaemia, the insulin dose should match both food intake and exercise, as both of these influence blood glucose levels (Hommel et al. 2017). This can be achieved either by a fixed insulin dose method, where individuals adjust their intake and activity according to the amount of insulin prescribed, or by a more flexible approach where insulin dose is adjusted according to meals and activities performed (Hommel et al. 2017). This method of insulin dose calculation is complex, as it involves estimating the carbohydrate content of the meal consumed, and an evaluation of factors that affect blood glucose levels such as stress, illness and activity (Hommel et al. 2017). Postprandial blood glucose levels play a significant role in glycaemic control and the aim is for patients to maintain a 2-h postprandial blood glucose level of less than 10 mmol/L (Fortin et al. 2017). To achieve this, patients need to adjust their insulin dose to the quantity of carbohydrate ingested (Fortin et al. 2017).

The ADA recognises that there is no one-size-fits-all approach to eating patterns and recommends that nutrition therapy should play an integral role in diabetes management (Evert et al. 2014). According to the ADA Standards of Diabetes Care, the newly diagnosed diabetic should be referred to a registered dietitian for nutrition therapy as soon as possible after diagnosis (Evert et al. 2014). In the United States of America (USA), only half of the people living with diabetes reported receiving diabetes education and less reported being seen by a dietitian (Evert et al. 2014). It is recommended that medical nutritional therapy (MNT) should include a nutrition assessment, nutritional diagnoses, nutrition interventions as well as nutrition monitoring and evaluation, which includes ongoing follow-up to support, evaluate and modify any changes, where needed (Evert et al. 2014). The registered dietitian is the preferred member of the healthcare team to provide MNT based on training, skills and expertise (Evert et al. 2014).

Dietary guidelines are available from the ADA, International Society for Pediatric and Adolescent Diabetes (ISPAD), Diabetes UK and the Society for Endocrinology, Metabolism and Diabetes of South Africa (SEMDSA) (ADA 2021b; Dyson et al. 2018; SEMDSA 2017; Smart et al. 2018). However, it is not known which of these dietary guidelines South African dietitians use in the management of T1DM, as there are currently no published dietary guidelines for the management of T1DM, specifically in South Africa. There is also a lack of published studies on the dietary practices used by dietitians in the management of T1DM. This study aimed to determine the dietary management practices for T1DM by dietitians in KwaZulu-Natal (KZN). KwaZulu-Natal was chosen as the study site because of the high prevalence of diabetes in the province. Approximately 34.1% of the KZN population is estimated to have diabetes (Sahadew, Singaram & Brown 2016).

## Methods

### Study design

This was a cross-sectional descriptive study.

### Study population

The study population included dietitians who were registered with the Health Professions Council of South Africa (HPCSA) and working in private and government settings within the province of KZN, at the time of the study. Dietitians who were completing their community service at the time of the study were excluded because of limited exposure to practice. Community service dietitians would have had less than 1 year of practice experience at the time of data collection. This was ensured by only allowing an option for more than 1 year of practice experience within the questionnaire itself. The KZN Department of Health (DOH) was approached through the National Institute for Health Research (NHIR) to invite dietitians employed by the DOH to participate in the study. The Association for Dietetics in South Africa (ADSA) in KZN was also approached to recruit its members to participate in the study. A total of 173 ADSA members and approximately 100 DOH-employed dietitians were eligible to participate in the study. Using Cochrane’s formula and assuming alpha = 0.05 and a margin of error of 0.05, the minimum sample required was calculated to be 160. However, only 67 responses were obtained. A number of dietitians started the survey but exited before completing it, resulting in a small sample size.

### Self-administered electronic questionnaire

A self-administered electronic questionnaire was developed for this study, as the study population was likely to have access to the Internet and would be able to access an electronic questionnaire. SurveyMonkey was used to distribute and manage the questionnaire. SurveyMonkey is an Internet-based survey programme that is useful in survey research (Ponto 2015). The questionnaire consisted mainly of close-ended questions and a 6-point Likert scale of agreement. The coding used for the Likert scale in the questionnaire was according to the agreement scale, where strongly disagree = 1, disagree = 2, slightly disagree = 3, slightly agree = 4, agree = 5 and strongly agree = 6. The ADA, ISPAD, Diabetes UK and SEMDSA guidelines for the management of diabetes were used to develop the questionnaire (ADA 2021b; Dyson et al. 2011; SEMDSA 2017; Smart et al. 2014).

An expert panel reviewed the questionnaire. This expert panel consisted of four dietitians with a special interest in diabetes and carbohydrate counting, working in the private, public and academic sectors. The expert panel assessed the questionnaire for appropriateness and comprehensiveness to meet the objectives. The questionnaire was revised according to their recommendations. The study supervisor and a statistician assured the content of the questionnaire by ensuring that the questions answered the research objectives of the study and that there was a logical flow with the questions without leading to any ambiguous or confusing questions. Consistency was ensured by providing subjects with clear and detailed instructions when completing the questionnaire and preventing questions from being skipped.

### Data collection

The link to the questionnaire was attached to an email, which was distributed to the dietitians who were members of the ADSA in KZN. The KZN DOH uploaded the survey on its intranet website under the surveys section, where the DOH dietitians could access the survey. As the DOH dietitians were not contacted personally, as with the ADSA (KZN) dietitians, permission was obtained from the Nutrition Directorate to contact the respective DOH hospitals where dietitians worked. These hospitals were contacted via email to alert them to the survey on the intranet.

### Data analysis

Data from the questionnaires were exported into a Microsoft Excel spreadsheet directly from SurveyMonkey. The data exported from SurveyMonkey were coded and checked for errors by the researcher. The data entry was cross-checked by a research assistant before being analysed by a statistician. Data were analysed by a statistician using the Statistical Package for Social Sciences (SPSS) version 26.0. Descriptive statistics was used to summarise the results. A chi-square goodness-of-fit test was used to identify response options selected significantly more than others in a categorical response variable with more than two categories. A binomial test was used to determine if a significant proportion selected one of the two possible responses. A one-sample *t*-test was used to test the average agreement against the central score of 3.5 to determine if there was significant agreement or significant disagreement when using the 6-point Likert agreement scale. The levels of agreement according to the 6-point Likert agreement scale were as follows: strongly disagree = 1, disagree = 2, slightly disagree = 3, slightly agree = 4, agree = 5 and strongly agree = 6. A significant result, with a mean score of greater or less than 3.5, implied significant agreement or disagreement, respectively. A *p*-value of < 0.05 was regarded as statistically significant.

### Ethical considerations

Ethical clearance was obtained from the Humanities and Social Science Ethics Committee of the University of KwaZulu-Natal (Reference number HSS/1612/018M). The KZN DOH gave approval for the study to be conducted on dietitians employed by the DOH. Permission was also obtained from ADSA (KZN) to conduct the study using its members. The dietitians were informed via a consent letter attached to the email wherein by opening the link to the survey, they were giving consent; however, they could opt out of the survey at any time.

## Results

### Sample characteristics

Sample characteristics are shown in Table 1. Just under 50% of the dietitians were 26–35 years of age. The majority (78.3%; *n* = 54) of dietitians attended the University of KwaZulu-Natal and 62.3% (*n* = 43) indicated that the Post Graduate Diploma in Dietetics was their highest qualification. Fifty-five per cent (*n* = 38) of dietitians indicated that they worked in the private sector, whilst 36.2% (*n* = 25) worked in the public sector and 8.7% (*n* = 6) worked in both the public and private sectors. More than 78% (*n* = 54) of the dietitians worked in an urban area, whilst 15.9% (*n* = 11) and 5.8% (*n* = 4) worked in semi-rural and rural areas, respectively. Sixty per cent (*n* = 42) of the dietitians were registered with the HPCSA for 1–10 years, with the mean being 10.9 years.

**TABLE 1:** Sample characteristics (*n* = 69).

Characteristic	Category	*n*	%
Age (years)	20–25	9	13.0
	26–35	33	47.8
	36–45	21	30.4
	46–55	4	5.8
	56–65	2	2.9
University attended	North-West University	2	2.9
	University of Cape Town	2	2.9
	University of KwaZulu-Natal	54	78.3
	University of Stellenbosch	6	8.7
	University of Pretoria	1	1.4
	University of the Western Cape	2	2.9
	Other	2	2.9
Highest qualification (*n* = 68)†	BSc Diet (Honours)	9	13.0
	BSc Diet	5	7.2
	PGDip Diet	43	62.3
	MSc Diet	10	14.5
	PhD	1	1.4
Sector of employment	Private	38	55.1
	Public	25	36.2
	Both private and public	6	8.7
Area of employment	Rural	4	5.8
	Semi-rural	11	15.9
	Urban	54	78.3
Number of years registered with the Health Professions Council of South Africa	1–10 years	42	60.9
	11–20 years	18	26.1
	21–30 years	6	8.7
	≥ 31 years	3	4.3

† , Some dietitians did not answer.

A significant number of initial appointments at diagnosis were at least 45 min long (30.4%, *n* = 21) (*p* = 0.001) and a significant number of initial appointments (34.8%; *n* = 24) (*p* = 0.001) were more than 1 h in duration. Although not statistically significant, 23.2% (*n* = 16) of patients were seen for a follow-up appointment at least once a month and 26.1% (*n* = 18) were seen less often than once a year. The majority of dietitians (87%; *n* = 60) used face-to-face methods for follow-up consultations (*p <* 0.05). Only 23.2% (*n* = 16) used email and 17.4% (*n* = 12) used the phone (Table 2).

**TABLE 2:** Background information on the management of type 1 diabetes mellitus (*n* = 62).†

Background information	Responses	*n*	%	p*
Average time spent with a patient who presents for the first time with a new diagnosis of T1DM	15 – < 30 min	3	4.3	**0.001**
	30 – < 45 min	14	20.3	
	45 min to 1 h	21	30.4	
	> 1 h	24	34.8	
Average frequency of follow-up visits for patients with T1DM	At least once a month	16	23.2	0.126
	At least once every 2 months	11	15.9	
	At least once every 6 months	11	15.9	
	At least once a year	6	8.7	
	Less often than once a year	18	26.1	
Methods used to review/follow-up patients with T1DM (*n* = 62)‡	Face-to-face	60	87.0	**< 0.05**
	Skype	0	0	
	Email	16	23.2	
	Phone	12	17.4	
	WhatsApp	2	2.9	

TIDM, type 1 diabetes mellitus.

† , Some dietitians did not answer.

‡ , Participants could select more than one option.

* , Chi-square goodness-of-fit test; *p*-values in bold are statistically significant.

### Background information on the management of type 1 diabetes mellitus

### Dietary guidelines used by dietitians

Dietary guidelines used by dietitians in the dietary management of diabetes are shown in Table 3.

**TABLE 3:** Dietary guidelines used by dietitians to manage type 1 diabetes mellitus (*n* = 57).†

Dietary guidelines	Response	*n*	%	p*
American Diabetes Association (ADA)	Yes	40	58.0	**0.003**
	No	17	24.6	
National Institute for Clinical Excellence (NICE)	Yes	15	21.7	**< 0.05**
	No	42	60.9	
Society for Endocrinology, Metabolism and Diabetes of South Africa (SEMDSA)	Yes	33	47.8	0.289
	No	24	34.8	
International Society for Pediatric and Adolescent Diabetes (ISPAD)	Yes	4	5.8	**< 0.05**
	No	53	76.8	
International Diabetes Federation (IDF)	Yes	16	23.2	**0.001**
	No	41	59.4	
European Association for the Study of Diabetes (EASD)	Yes	3	4.3	**< 0.05**
	No	54	78.3	
Other	Yes	5	7.2	-
	No	52	75.4	

† , Some dietitians did not answer.

* , Binomial test; *p*-values in bold are statistically significant.

Approximately 58% (*n* = 40) of the dietitians used the ADA guidelines as a resource (*p* = 0.003), whilst a significant number (60.9%; *n* = 42) indicated that they did not use the NICE guidelines. The other guidelines that were not used by dietitians included the ISPAD guidelines (76.8%; *n* = 53) (*p <* 0.05), IDF (59.4%; *n* = 41) (*p* = 0.001) and the EASD (78.3%; *n* = 54) (*p <* 0.05) (Table 3).

### Dietary approaches used or recommended when treating type 1 diabetes mellitus

The dietary approaches used or recommended when treating patients with T1DM are presented in Table 4.

**TABLE 4:** The dietary approaches used or recommended when treating patients with type 1 diabetes mellitus (*n* = 58).†

Dietary approaches	Strongly disagree		Disagree		Slightly disagree		Slightly agree		Agree		Strongly agree		Mean agreement score	p*
	*n*	%	*n*	%	*n*	%	*n*	%	*n*	%	*n*	%		
Glycaemic index	2	2.9	3	4.3	3	4.3	12	17.4	28	40.6	10	14.5	4.57	**< 0.05**
Portion control using the healthy eating plate	0	0	3	4.3	1	1.4	3	4.3	27	39.1	24	34.8	5.17	**< 0.05**
Carbohydrate counting using scales and weighing items	4	5.8	16	23.2	11	15.9	15	21.7	10	14.5	2	2.9	3.29	0.239
Carbohydrate counting using nutritional labels	1	1.4	6	8.7	5	7.2	17	24.6	25	36.2	4	5.8	4.22	**< 0.05**
Carbohydrate counting using household measures	0	0	3	4.3	4	5.8	11	15.9	30	43.5	10	14.5	4.69	**< 0.05**
Carbohydrate awareness, that is, making patients aware of which foods contain carbohydrate	0	0	0	0	0	0	1	1.4	15	21.7	42	60.9	5.71	**< 0.05**

† , Some dietitians did not answer.

* , One-sample *t*-test; *p*-values in bold are statistically significant.

There was significant agreement that dietitians used or recommended the following approaches when treating T1DM: glycaemic index, portion control using the healthy eating plate, carbohydrate counting using nutritional labels, carbohydrate counting using household measures, carbohydrate awareness, that is, making patients aware of which foods contain carbohydrate (*p <* 0.05 in each case) (Table 4).

### Resources used in the dietary management of type 1 diabetes mellitus

Dietitians were asked to state which resources they used to assist patients in the dietary management of T1DM. The resources used to assist patients in the dietary management of T1DM are presented in Table 5.

**TABLE 5:** Resources used in the dietary management of type 1 diabetes mellitus.

Resources	Frequency of use	% frequency†	p*
Healthy eating plate	49	71.0	**< 0.05**
Exchange lists	29	42.0	1.000
Household measures	51	73.9	**< 0.05**
Food models	31	44.9	0.694
Pictorial guide	27	39.1	0.694
Other	6	8.7	-

† , Participants could select more than one option.

* , Binomial test; *p*-values in bold are statistically significant.

A significant number of dietitians indicated that they used the ‘healthy eating plate’ (71%; *n* = 49) (*p <* 0.05) and ‘household measures’ (73.9%; *n* = 51) (*p <* 0.05) to assist patients in the dietary management of T1DM (Table 5). Other resources mentioned included actual food products with their labels, food photos, practical weighing in the clinic with real food samples, individualised meal plans that are used in state hospitals, pictures of food items and an individualised eating plan that contains a selection of quantified starch and protein choices, practical application exercise and the block system for carbohydrate counting.

### Factors that influenced the choice of dietary management approach

Dietitians were asked to indicate their level of agreement regarding the factors that influenced their choice of dietary management approach using a six-point Likert agreement scale (Table 6).

**TABLE 6:** Factors that influenced the choice of dietary management approach (*n* = 58).†

Factors	Strongly disagree		Disagree		Slightly disagree		Slightly agree		Agree		Strongly agree		Mean agreement score	p*
	*n*	%	*n*	%	*n*	%	*n*	%	*n*	%	*n*	%		
Time constraints	1	1.4	5	7.2	6	8.7	17	24.6	12	17.4	17	24.6	4.47	**< 0.05**
Literacy level of patient	0	0	0	0	5	7.2	5	7.2	15	21.7	33	47.8	5.31	**< 0.05**
Resources available	0	0	6	8.7	6	8.7	5	7.2	17	24.6	24	34.8	4.81	**< 0.05**
Language barrier	4	5.8	8	11.6	3	4.3	9	13.0	12	17.4	22	31.9	4.43	**< 0.05**

† , Some dietitians did not answer.

* , One-sample *t*-test; *p*-values in bold are statistically significant.

Results from a one-sample *t*-test showed that there was a strong significant agreement that time constraints, the literacy level of the patient, available resources and language barriers all played a role in determining the choice of dietary management approach by dietitians (*p <* 0.05 in each case) (Table 6).

## Discussion

This study aimed to determine the dietary practices used by dietitians in KZN to manage T1DM. About 23% of dietitians indicated that patients attended follow-up visits ‘at least once a month’, which is in line with the SEMDSA (2017) recommendation of three to four visits within the first 3–6 months of diagnosis. There are currently no published dietary guidelines for the management of T1DM in South Africa and it is not known which dietary guidelines KZN dietitians currently use in the management of T1DM. This study found that more dietitians used the ADA guidelines, which are from the USA, compared to the local SEMDSA guidelines. Guidelines that specifically include T1DM and the dietary management thereof include ISPAD, EASD and NICE. However, these guidelines were not widely used by the dietitians in this study. This indicates that there is a need for guidelines on the dietary management of T1DM specific to South Africa, as the population in South Africa differs to that of other countries including the USA (Owolabi et al. 2018).

Ongoing follow-up and support are key to the successful management of diabetes and this could be in the form of face-to-face visits, peer support, phone coaching or through social media platforms (Maryniuk, Evert & Rizzotto 2019:481). The majority of dietitians indicated that they used face-to-face methods to review patients, whilst Skype consultations were not used at all by the dietitians as a means of follow-up. The reasons for this could be because of the lack of access to technology as well as patients being uncomfortable with the use of technology. Some of the additional reasons for the low use of phone and email for follow-up consultations could be because of the lack of access to technology by both patients and dietitians, and the lack of access to a work phone and the cost of the use of the phone (Piette 2007). There is a paucity of evidence regarding the use of Skype and the risks and benefits it poses for healthcare professionals and their patients. Further studies are therefore warranted to understand the potential role that Skype consultations could play in telemedicine (Armfield, Gray & Smith 2012). Dietitians should consider other modes of follow-up as a potential way to deliver a service and they should increase their knowledge and competency in the area of telemedicine (Dalton 2008).

The GI was one of the main dietary approaches used to manage diabetes in the current study. This could be because of the fact that the there are numerous foods which have been endorsed by the Glycaemic Index Foundation of South Africa (GIFSA). The logo appears on the label of specific foods, indicating the GI of the product and how frequently the food can be consumed. Dietitians could be using the GIFSA logos on food labels to teach and guide their diabetic patients in terms of food choices. There was a significant use of carbohydrate counting using nutritional labels in the private sector and foods with the GIFSA logos could be a part of that group of foods. Another possible reason for the wide use of the GI could be that the GI concept has been described in the literature since the 1980s (Grant & Wolever 2011). Although it is a controversial dietary management method, dietitians are instrumental in assisting patients to understand and apply the concept to their diabetes management (Grant & Wolever 2011). A group of South African dietitians (*n* = 36) attending a Masters class agreed that there are proven beneficial effects of a low GI diet versus a high GI diet, in the prevention and treatment of T2DM (Vorster 2005). However, there is conflicting evidence on the effectiveness of the GI as a meal planning approach (Franz et al. 2010). In a South African study, by investigating the dietary management practices of dietitians for T2DM in public hospitals in the Limpopo province, information on nutrition education materials was collected (Ceronio & Mbhenyane 2017). Results from 21 different hospitals highlighted that ‘general guidelines and a foods allowed/avoided list’ were the most common education materials used. Only two dietitians responded that they used a ‘glycaemic index’ sheet (Ceronio & Mbhenyane 2017). This was contrary to the current study, which found that the GI was one of the main dietary management approaches used.

In the current study, portion control using the healthy eating plate was widely used. The plate model is a type of eating pattern that has been found to be an effective and useful method of controlling carbohydrate intake in diabetics (Maryniuk 2017). It is a simplified way of teaching patients without using lists of foods and confusing calculations, but it still takes healthy eating into consideration (Maryniuk 2017; Maryniuk et al. 2019:481). If individuals require guidelines that are more specific, then issuing individual guidelines per meal could be implemented (Maryniuk et al. 2019:481). Additional reasons for the dietitian’s preference for the use of the plate model and household measures could be that these resources are more readily available in both the private and public sector as they are cheap and simple to explain and understand (Gray & Threlkeld 2019). Bowen et al. (2016) concluded from their study on patients with T2DM that the use of carbohydrate counting and a modified plate method by certified diabetes educators to educate patients, both improved glycaemic control. The modified plate model may be better accepted as opposed to carbohydrate counting in patients with varied numeracy skills. However, further research is required to make a direct comparison of these two approaches (Bowen et al. 2016). In the current study, dietitians used nutritional labels, carbohydrate awareness and household measures as their main approach to carbohydrate counting. Although dietitians agreed that they taught their patients to read the total carbohydrate content from nutritional labels of food products, it was unclear which dietary management method they used alongside this approach. According to Smart et al. (2009), there is no clear evidence as to which method of estimation is better. The current study found that exchange lists were less frequently used by dietitians in the dietary management of T1DM. This could be because of low patient literacy levels and language barriers. According to Pastors et al. (2005:201), exchanges lists are effective in nutrition education but their use is dependent on the needs and abilities of the individual patient.

There was a strong agreement amongst the dietitians that patient’s literacy levels played a role in determining the choice of dietary approach used. According to a study assessing health literacy and numeracy of patients in diabetes care, it was found that the lack of these skills is not always obvious to the educator. Assessment of these areas is key in improving the education experience for both patient and provider whilst also improving clinical outcomes (White et al. 2010). Assessment of literacy and numeracy should possibly be included in the dietitian’s initial assessment of the patient. Only 30% of people with diabetes from a 2014 survey said that they felt ‘totally confident’ in their ability to eat as per recommendations (Maryniuk 2017). This highlights the important role that dietitians have in educating and empowering patients in the dietary management of their diabetes.

### Study limitations and recommendations

A major limitation of this study is the low participation rate. A number of dietitians started the questionnaire and exited before completing it, possibly because they were not involved in the dietary management of T1DM. There is no register indicating which dietitians treat T1DM, so it was not possible to identify relevant dietitians for the study. Not all dietitians who work in KZN are members of ADSA or work for the DOH. Therefore, it is possible that some dietitians in KZN were not invited to participate in the study. The sample was therefore not a true representation of all the dietitians in the province of KZN and this prevents generalised conclusions from being drawn. Because an electronic on-line survey was used, it is possible that the participant who answered it was an unintended recipient of the survey. It is also possible that the participant looked up the answers to the survey or allowed someone else to answer the questions for them. All participants of the study took part voluntarily. The fact that the study relied on volunteers could have created bias and affected the size of the sample. It was possible that by addressing T1DM specifically in the study, it may have limited the number of responses received. This study highlights the need for further investigation of whether the guidelines used to manage T1DM are suitable or should be adapted to treat South African patients specifically. A larger study incorporating all dietitians in South Africa should be conducted and participants should be recruited from both the private and public sectors.

## Conclusion

Most dietitians in this study used the ADA dietary guidelines to manage T1DM. However, guidelines from the USA may not be the most appropriate for diabetics in South Africa. This highlights the need for South African guidelines for the dietary management of T1DM. Most dietitians spent between a quarter of an hour to more than an hour with patients presenting for the first time with a new diagnosis of T1DM and used face-to-face methods for follow-up consultations. Dietary approaches used or recommended when treating patients with T1DM included the glycaemic index, portion control using the healthy eating plate, carbohydrate counting using nutritional labels, carbohydrate counting using household measures and carbohydrate awareness. Household measures and the healthy eating plate were the resources most used in the dietary management of T1DM. Training regarding the use of resources should be incorporated when training dietitians on the dietary management of DM. Time constraints, the literacy level of the patient, available resources and language barriers all played a role in determining the choice of dietary management approach by dietitians. It can be concluded that dietitians in KZN used different practices for the dietary management of T1DM.
